# Evaluating the Application Potential of a Recombinant *Ganoderma* Protein as Bioactive Ingredients in Cosmetics

**DOI:** 10.3390/molecules28073272

**Published:** 2023-04-06

**Authors:** Zhi-Jian Guo, Yan Liu, Jia-Yi Yang, Meng-Yuan Jin, Pei-Wen Mao, Xuan-Wei Zhou

**Affiliations:** School of Agriculture and Biology, Engineering Research Center of Therapeutic Antibody (Ministry of Education), Shanghai Jiao Tong University, Shanghai 200240, China; gzj19991006@sjtu.edu.cn (Z.-J.G.); liuyan8468@sjtu.edu.cn (Y.L.);

**Keywords:** recombinant FIP-glu (rFIP-glu), antioxidant activity, whitening potency, melanin, tyrosinase

## Abstract

The aim of this study was to evaluate the application potential of a recombinant fungal immunomodulatory protein from *Ganoderma lucidum* (rFIP-glu). First, a recombinant plasmid pPIC9K::FIP-glu-His was transferred into *Pichia pastoris* for the production of protein. The protein was then to assess its free radical scavenging abilities and the effect on the viability of both human immortalized keratinocytes (HaCaT cells) and mouse B16-F10 melanoma cells (B16 cells) *in vitro*, followed by the effect on the melanin synthesis of B16 cells. The results of SDS-PAGE and western blot showed that rFIP-glu was successfully expressed. Furtherly, a bioactivity assay *in vitro* indicated that the scavenging rate of 2,2-diphenyl-1-picrylhydrazyl (DPPH) radicals reached 84.5% at 6.0 mg/mL (*p* ≤ 0.0001) of rFIP-glu, showing strong antioxidant activity. Subsequently, a safety evaluation demonstrated that rFIP-glu promoted the proliferation of HaCaT cells, with the cell viability reaching 124.3% at 48 μg/mL (*p* ≤ 0.01), regarding the cell viability of B16 cells after exposure to rFIP-glu (48 μg/mL) significantly inhibited, to 80.7% (*p* ≤ 0.01). Besides, rFIP-glu inhibited the melanin synthesis of B16 cells in a dose-dependent manner from 100–1000 μg/mL, and rFIP-glu at 500 μg/mL (*p* ≤ 0.01) exhibited the highest intracellular melanin amount reduction of 16.8%. Furthermore, a mechanism analysis showed that rFIP-glu inhibited tyrosinase (TYR) activity by up-regulating the expression of the microphthalmia-associated transcription factor (MITF) and down-regulating the gene expression of TYR and tyrosinase-related protein-1 (TYRP-1), thus inhibiting melanin synthesis. The data implied that rFIP-glu had significant antioxidant activity and whitening potency. It should be used as raw materials for cosmeceutical applications.

## 1. Introduction

*Ganoderma lucidum* (Lingzhi or *Ganodrema*), a precious medicinal mushroom, has been documented as possessing a variety of pharmaceutical functions in several Chinese classics. The property of “toning up the skin” has led to the belief that the cosmetic potential of *G. lucidum* would be exploited. Currently, *Ganoderma* extracts (including *G. lucidum*, *G. atrum* and *G. sinensis*) have been listed in the catalog of cosmetic ingredients available for usage in China. *Ganoderma* is rich in active ingredients such as polysaccharides, terpenoids, and proteins, etc. Among them, polysaccharides and terpenoids are mostly mixtures whose activities often differ significantly depending on their extraction methods [1], and that are unsuitable for the quality control of preparations. Functional protein is popular in the medical and food fields due to its stable composition and well-defined structure. Fungal immunomodulatory proteins (FIPs) are a subclass of active ingredients isolated from *Ganoderma* spp. and have a well-defined structure in addition to therapeutic effects. However, the content of FIPs is low in natural mushrooms. Evidence has shown that natural FIP-glu derived from *G. lucidum* gave only a yield of 5–10 mg of purified FIP-glu per 300 g mycelium [2]. Fortunately, with the development of biotechnology, the utilization of biotechnology to increase the production FIPs shows great potential for application [3,4].

Melanin, a pigment commonly found in animals, plants and humans, is prominent in the skin, mucosa, retina, and ovaries. Melanin is synthesized by specialized pigment-producing cells known as melanocytes through a series of enzymatic and spontaneous reactions related to tyrosine conversion [5], accompanied by the production of reactive oxygen species (ROS) and reactive oxidants [6]. Melanosomes within melanocytes enable the synthesis of melanin that was transferred to the surrounding keratinocytes, providing the basic tone of human skin [7]. One of the main functions of melanin is to absorb ultraviolet (UV) and markedly reduce the damage of UV to DNA and prevent skin diseases [8]. However, if excessive melanin secretion cannot be metabolized by the body in a timely fashion, it will deposit on the surface of the skin and form black spots. The increase of skin pigmentation is a problem that cannot be ignored, and is secondary to a variety of factors, such as age, endocrine disorders, hormone levels, inflammation, skin diseases caused by UV, and infrared radiation [9]. The deposition of melanin in the epidermis leads to skin pigmentation [10], which affects the physical appearance and causes lesions such as nevus or melanoma. Hence, ROS scavenging and modulate melanogenesis inhibiting have attracted greater attention of late.

In this study, the rFIP-glu gene was overexpressed in *Pichia pastoris* to improve rFIP-glu production, and the antioxidant potential was first evaluated. Next, the cell viabilities of HaCaT cells and B16 cells, and the B16 cells’ morphology underwent a safety evaluation for the rFIP-glu used in cosmetics. Subsequently, the antioxidant capacity and melanogenesis of rFIP-glu were assessed to evaluate the value of its application as a supplement in whitening cosmetics. Furthermore, the tyrosinase (TYR) activity and its related genes’ expression were routinely investigated, which was employed to explain the melanogenesis-inhibitory effect of rFIP-glu and its action mechanism.

## 2. Results and Discussion

### 2.1. Preparation of rFIP-glu

**Figure 1:**
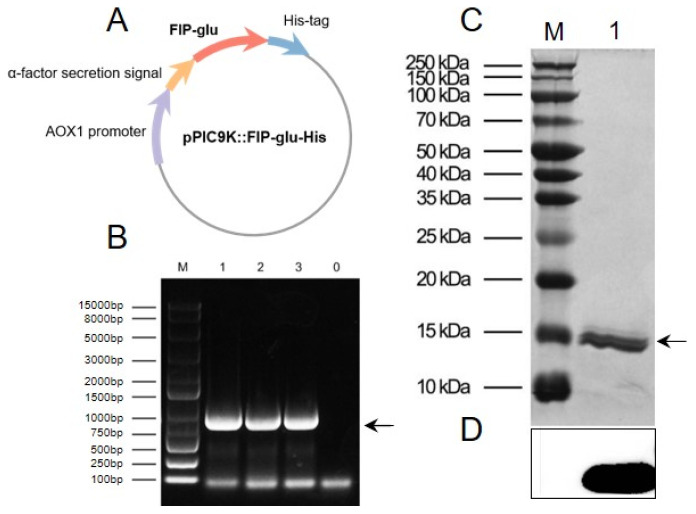
Preparation of rFIP-glu in *Pichia pastoris*. (**A**) Construction of the recombinant plasmid pPIC9K::FIP-glu-His. (**B**) Identification of yeast transformants by PCR analysis. Lane M, DNA Ladder; Lane 1–3, rFIP-glu yeast transformant DNA; Lane 0, negative control. (**C**) SDS-PAGE analysis. Lane M, molecular weight marker; Lane 1, rFIP-glu. (**D**) Western blot analysis.

The recombinant plasmid pPIC9K::FIP-glu-His (Figure 1A), constructed on our laboratory platform with a His-tag, was employed to express the recombinant protein rFIP-glu in *Pichia pastoris*. The linearized recombinant plasmid was transferred into the *P. pastoris* GS115 stain, subject to yeast genomic DNA extraction, and identified by PCR. As shown in Figure 1B, the yeast transformants exhibited specific bands between 800–900 bp (theoretical value of 847 bp), indicating that the recombinant plasmid had been successfully transferred into *P. pastoris*. An SDS-PAGE analysis of a protein of about 15 kDa (Figure 1C) and western blot analysis of the recombinant protein rFIP-glu containing 6× His-tag (Figure 1D) demonstrated that rFIP-glu was effectively expressed. So far, a series of FIPs sequences cloned from *G. lucidum*, such as FIP-glu, FIP-gts and FIP-gmi, were effectively expressed in bacteria, fungi or other cells [11]. *P. pastoris* is a biological expression system, and enables its transformants to express exogenous proteins stably and modifies protein glycosylation, and is therefore suitable for industrial production [12]. In the present study, rFIP-glu produced by *P. pastoris* GS115 was identified similar to the protein from *G. lucidum.*

### 2.2. Cytotoxicity of rFIP-glu on HaCaT and B16 Cells

**Figure 2:**
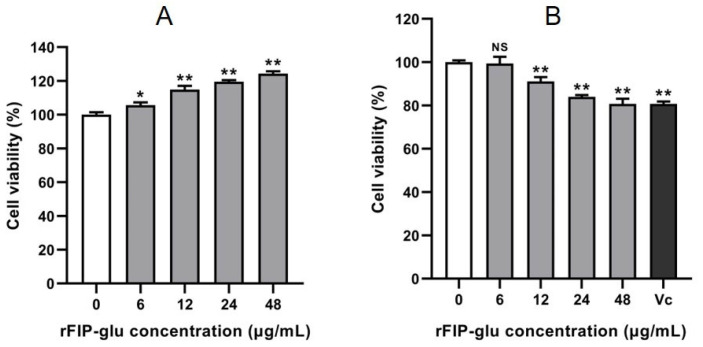
Safety evaluation. The effects of rFIP-glu on the cell viability of (**A**) HaCaT cells and (**B**) B16 cells. Vc (40 μg/mL) was used as the positive control. Data are expressed as means ± SD (n = 3). NS, *p* > 0.05; *, *p* ≤ 0.05; **, *p* ≤ 0.01 versus the control group.

Currently, compounds such as arbutin, kojic acid and vitamin C (Vc) have been used for de-pigmentation, but the demand for alternative active ingredients from natural sources with skin benefits and less toxicity is still strong [13]. Meanwhile, the efficacy evaluation of these ingredients is an important step in the development process [14]. HaCaT cells are extensively employed in a safety evaluation and in the antioxidant efficacy assessment of cosmetic products [15,16]. A cytotoxicity assay was performed to evaluate the safety of rFIP-glu as a cosmeceutical formulation before exploring the efficacy of rFIP-glu as an active ingredient acting on dermal cells. Figure 2A showed that rFIP-glu had a significant promotion effect on the cell viability of HaCaT cells within the range of 6–48 μg/mL in a dose-dependent manner, and the cell viability of HaCaT cells reached 124.3% at 48 μg/mL (Figure 2A; *p* ≤ 0.01). This result suggests that rFIP-glu at 6–48 μg/mL had no cytotoxicity to human HaCaT cells.

With regard to B16 cells, rFIP-glu inhibited the growth at 12–48 μg/mL in a dose-dependent manner, and the cell viability by rFIP-glu treatment was 91.1% to 80.7% (Figure 2B; *p* ≤ 0.01). Meanwhile, as a positive control, Vc (40 μg/mL) treatment led to 80.7% cell viability of the B16 cells. Therefore, rFIP-glu within the range of 12–48 μg/mL had low cytotoxicity for B16 cells. Melanocytes located in the epidermis and hair follicles have significant proliferation and development ability and are an important source of melanin [17,18]. Pigmented melanoma cell lines and melanocytes have often been used to assess melanogenesis regulators in vitro [19,20]. The amount of melanin synthesis was closely related with the number and cell activity of melanocytes. Although rFIP-glu exhibited a certain inhibitory effect on the growth of B16 cells, the cell viability was maintained at about 80% under 48 μg/mL treatment, which met the basic demand for cosmetic safety evaluation at the cytotoxicity level. This is the first study to evaluate the safety of a cosmeceutical formulation supplemented with *Ganoderma* protein.

### 2.3. Cell Morphology and the Apoptosis of B16 Cells

**Figure 3:**
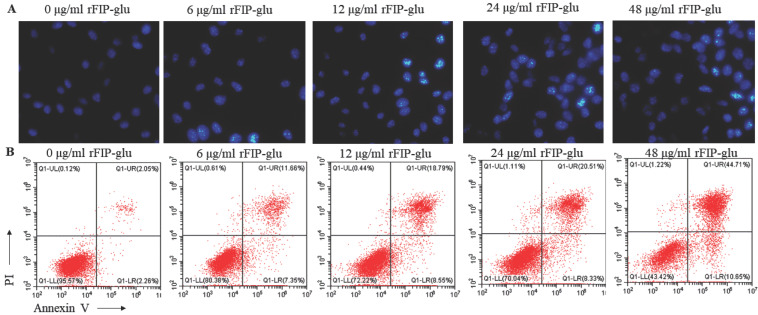
rFIP-glu inducing the apoptosis of B16 cells. (**A**) Hoechst 33258 staining of B16 cells under a fluorescence microscope (400×). (**B**) B16 cells stained by Annexin/PI and analyzed by flow cytometry.

In order to investigate the effect of rFIP-glu on the morphology of B16 cells, Hoechst 33258 staining solution was used to stain B16 cells treated with different concentrations of rFIP-glu (0, 6, 12, 24, 48 μg/mL). Under a fluorescence microscope, the control group cells were spindle-shaped and evenly stained. As the rFIP-glu concentration increased, the B16 cells became swollen. In the meanwhile, the nuclear group became shrunk, pyknotic, fragmented, and the density of nuclear chromatin increased gradually (Figure 3A). The Hoechst 33258 staining solution is a blue fluorescent dye that can penetrate the cell membrane, and is often used for the detection of apoptosis and ordinary nuclear staining. After staining, the density of the nuclear chromatin of apoptotic cells, which are half-moon or horseshoe in shape, will be increased. Obviously, B16 cells treated with rFIP-glu showed a morphology that is characteristic of apoptosis. Hence, it was reasonably assumed that rFIP-glu promoted the apoptosis of B16 cells. Next, the rate of apoptosis in B16 cells treated with rFIP-glu (0, 6, 12, 24, 48 μg/mL) was determined by flow cytometry. As shown in Figure 3B, the rate of apoptosis increased from 11.56% to 44.71%, while the rate of apoptosis without rFIP-glu treatment was 2.05%. These results indicated that rFIP-glu indeed induced the apoptosis of B16 cells.

### 2.4. Antioxidant Activity

**Figure 4:**
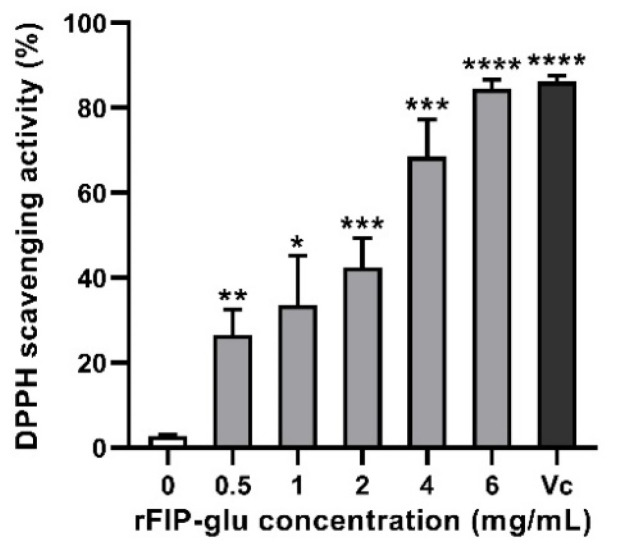
The effect of rFIP-glu on the scavenging rate of DPPH radicals. Vc (0.5 mg/mL) was used as the positive control. Data are expressed as means ± SD (n = 3). *p* > 0.05; *, *p* ≤ 0.05; **, *p* ≤ 0.01; ***, *p* ≤ 0.001; ****, *p* ≤ 0.0001 versus the control group.

The antioxidant activity of rFIP-glu was characterized according to the DPPH radical scavenging capacity. The result showed that DPPH radicals exhibited a marked upward trend (IC_50_ value = 2.18 mg/mL) at the indicated concentrations of rFIP-glu (Figure 4). The DPPH radical scavenging capacity reached 68.6% at 4.0 mg/mL (*p* ≤ 0.001) and 84.5% at 6.0 mg/mL (*p* ≤ 0.0001) of rFIP-glu treatment, respectively, indicating that rFIP-glu had a strong antioxidant activity compared to the control group (Figure 4). A previous study demonstrated that the higher antioxidant activity of peptide/protein might be attributed to the higher content of aromatic amino acids, due to its structure being similar to polyphenols, which are capable of donating protons to electron-deficient free radicals and preventing damage caused by sebum oxidative stress [21]. As reported by Kino et al. [2], the aromatic amino acid content of rFIP-glu was 13.9% (Phe 5.98%, Tyr 5.23%, Trp 2.64%), which contributed to the considerable antioxidant activity. Furthermore, the high content of aspartic acid (Asp) in rFIP-glu was considered to be a stabilizer of vitamin E in cosmeceuticals against UV damage and skin cancer [2]. Although the antioxidant capacity of rFIP-glu is slightly weaker compared to Vc (an IC_50_ value = 0.21 mg/mL), its advantage lies in the more diverse potency potential shown in its availability for use in cosmetics.

### 2.5. Melanin Synthesis

**Figure 5:**
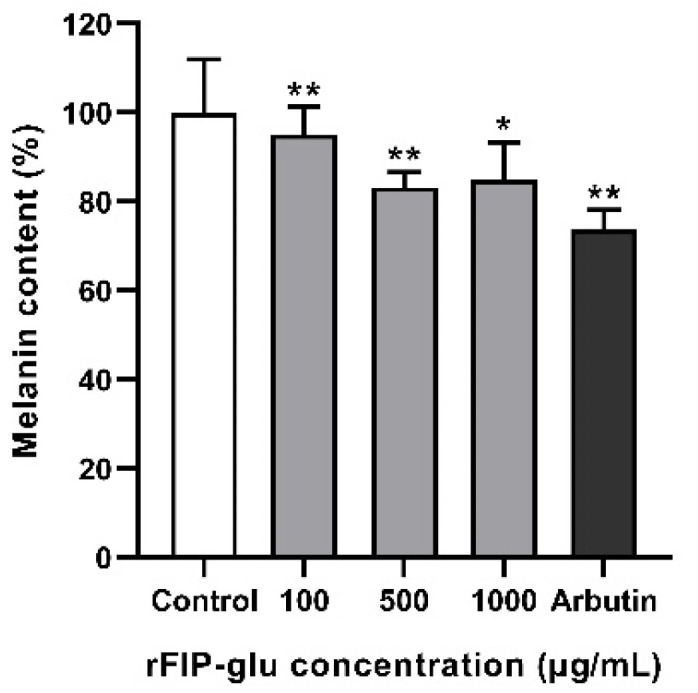
Effect of rFIP-glu on the melanin content of B16 cells. Arbutin (100 μg/mL) was used as the positive control. Data are expressed as means ± SD (n = 3). *, *p* ≤ 0.05; **, *p* ≤ 0.01 versus the control group.

To investigate the effect of rFIP-glu on melanin synthesis, B16 cells were treated with rFIP-glu (0, 100, 500, 1000 μg/mL). Arbutin at 1000 μg/mL served as a positive control. The results showed that rFIP-glu exhibited inhibitory effects on melanogenesis at 500 μg/mL (*p* ≤ 0.01) and 1000 μg/mL (*p* ≤ 0.05), and rFIP-glu (500 μg/mL) treatment led to a melanin synthesis reduction of 16.8% (Figure 5). The long-term exposure to UV will make skin cells release melanin, which can effectively absorb ultraviolet rays to reduce the damage, and finally melanin will be metabolized by the body. However, when the metabolism of the body is poor, part of the melanin will deposit on the skin surface. Some ROS scavengers and inhibitors that inhibit UV-induced melanogenesis were used to treat hyperpigmented spots of skin, such as Vc, arbutin, and kojic acid [22,23]. Melanin synthesis and tyrosinase activity are important indexes to evaluate the whitening efficacy of cosmeceutical ingredients [24,25]. Arbutin, a polyphenolic compound derived from various plant metabolites, is one of the most widely used ingredients supplemented in cosmetics for skin-whitening and skin-care because of its ability to prevent the accumulation of melanin in skin [26]. In the present study, the inhibitory effect of rFIP-glu on melanin synthesis was almost equal to that of arbutin (Figure 5; *p* ≤ 0.01). Hence, rFIP-glu was one kind of bioactive *Ganoderma* protein for depigmentation, and showed a good skin-whitening potential.

### 2.6. Tyrosinase Activity and mRNA Expression of Related Genes

**Figure 6:**
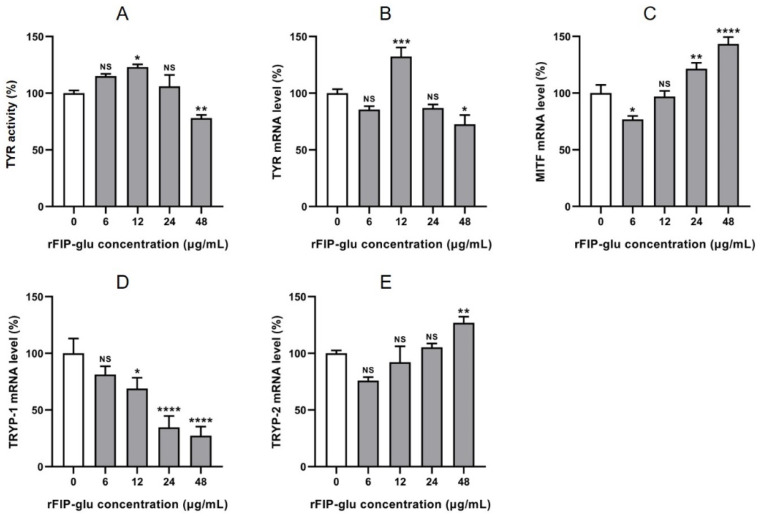
The effects of rFIP-glu on tyrosinase activity and mRNA expression of related genes in B16 cells. Tyrosinase activity (**A**); mRNA expression of TYR (**B**), MITF (**C**), TYRP-1 (**D**), and TYRP-2 (**E**). Data are expressed as means ± SD (n = 3). NS, *p* > 0.05; *, *p* ≤ 0.05; **, *p* ≤ 0.01; ***, *p* ≤ 0.001; ****, and *p* ≤ 0.0001 versus the control group.

Tyrosinase (TYR) is the key rate-limiting enzyme in the process of melanogenesis, and TYR activity strongly affects the entire pigmentation process [27]. The higher activity of TYR could lead to a higher amount of melanogenesis. To investigate the mechanism of melanogenesis inhibition of rFIP-glu, TYR activity was firstly detected after B16 cells were treated with different concentrations (0, 6, 12, 24, 48 μg/mL). Figure 6A showed that rFIP-glu at 48 μg/mL significantly inhibited TYR activity in B16 cells, and TYR activity was 76.50% (*p* ≤ 0.01) compared with the control group. The low concentration of rFIP-glu had no inhibitory effect on TYR activity. Because of the importance of TYR in melanogenesis, the direct inhibition of TYR activity is the most vital target of melanogenesis inhibition. Currently, the most commercially available skin whitening agents are TYR inhibitors [28].

Apart from TYR, melanogenesis is initiated and regulated by many signal systems and transcription factors, such as MITF, TYRP-1 and TYRP-2 [29,30]. The mRNA expression of related genes during melanin synthesis was investigated in B16 cells. The results showed that the mRNA expression of TYR was inhibited by 48 μg/mL rFIP-glu (Figure 6B; *p* ≤ 0.05). The mRNA expression of MITF was inhibited at 6 μg/mL rFIP-glu (Figure 6C; *p* ≤ 0.05), while it was promoted by 24 and 48 μg/mL rFIP-glu (Figure 6C; *p* ≤ 0.01 at 24 μg/mL and *p* ≤ 0.0001 at 48 μg/mL). rFIP-glu at 6–48 μg/mL had a significant inhibitory effect on the mRNA expression of TYRP-1 in a dose-dependent manner (Figure 6D). In addition, the low concentration of rFIP-glu had no significant effect on TYRP-2 mRNA expression, except that 48 μg/mL rFIP-glu promoted the expression. These results indicate that rFIP-glu affected the TYR activity by down-regulating the mRNA expression of TYR and TRYP-1, and up-regulating the mRNA expression of MITF, which further affected the melanogenesis in B16 cells. These results were slightly different with the previous publication by Jeon et al. [30], who’s study showed that leaf skin extracts of Aloe vera fermented by *Lactobacillus plantarum* BN41 had skin-lightening effects with decreasing melanogenesis for the down-regulating gene expression of MITF, TYR, TYRP-1 and TYRP-2 (*p* ≤ 0.05). It has been reported that MITF plays important roles in the regulation of melanogenesis by regulating the genes expression of melanogenic enzymes, including TYR, TYRP-1, and TYRP-2 [31,32]. In general, our present study showed that rFIP-glu functioned by initially stimulating levels of MITF and inhibiting the TYRP-1/TYR pathway, which thereby inhibited tyrosinase activity and melanogenesis. Therefore, it is essential to regulate the TYR activity and the genes expression associated with melanogenesis in order to treat the hyperpigmentation of the skin.

## 3. Materials and Methods

### 3.1. Strain, Cells and Reagents

The recombinant plasmid pPIC9K::FIP-glu-His and *Pichia pastoris* GS115 strain were preserved in our laboratory. HisSep nickel-nitrilo-triacetic acid (Ni-NTA) agarose resin was purchased from Yeasen (Shanghai, China). The restriction enzyme Sac I was purchased from Takara (Beijing, China). DPPH and vitamin C were purchased from Innochem (Beijing, China). The CCK-8 kit and horseradish peroxidase (HRP)-conjugated goat anti-mouse IgG and the Bradford protein assay kit were procured from Sangon (Shanghai, China). Other reagents were of analytical grade.

### 3.2. Preparation of rFIP-glu

The expression system of *Pichia pastoris* GS115 with a recombinant plasmid pPIC9K::FIP-glu-His was employed for producing rFIP-glu according to the previous method [33]. Briefly, the recombinant plasmid pPIC9K::FIP-glu-His was linearized by the restriction enzyme *Sac I,* and then transferred into *P. pastoris* GS115. The yeast transformants were fermented in Yeast Peptone Dextrose (YPD) and induced by the expression of methanol. The yeast transformants were initially cultured in Yeast Peptone Dextrose Medium (YPD), while the carbon source was later removed by Buffered Glycerol-complex Medium (BMGY) and finally methanol-induced fermentation in Buffered Methanol-complex Medium (BMMY) was performed to produce rFIP-glu. The above fermentation broth was then collected, subjected to bacterial precipitation, and diluted in a lysis buffer (50 mM NaH_2_PO_4_, 300 mM NaCl, 10 mM imidazole, pH 8.0) for liquid exchange. The recombinant proteins were purified with HisSep Ni-NTA agarose resin, and dialysis and lyophilization were then performed. The dried protein fractions were dissolved in a PBS solution, followed by the identification via sodium dodecyl sulfate-polyacrylamide gel electrophoresis (SDS-PAGE) and western blot analysis.

### 3.3. Cell Culture

B16 cells and HaCaT cells were purchased from the Cell Bank of the Chinese Academy of Sciences (Shanghai, China), cultivated in Dulbecco’s Modified Eagle’s Medium (DMEM) high glucose medium (Gibco, Grand Island, NY, USA), supplemented with antibiotics (100 U/mL penicillin and 100 mg/mL streptomycin) and 10% fetal bovine serum (FBS) (Gibco, Grand Island, NY, USA), and incubated at 37 °C and 5% CO_2_ in an incubator. The cells were seeded at 4 × 10^4^ cells per well in 12- or 96-well microplates.

### 3.4. Cell Viability Assay

The cell viability of HaCaT and B16 cells was assessed by a CCK-8 assay after the cells were treated with rFIP-glu. HaCaT and B16 cells were seeded in a 96-well plate with a density of 4 × 10^4^/mL, respectively. After incubation for 24 h, the culture was replaced with different concentrations of rFIP-glu (0, 6, 12, 24, 48 μg/mL) and incubated for 24 h. A 10 μL CCK-8 solution was added and incubated for 0.5–1 h. The absorbance was measured at 450 nm by the microplate reader (BioTek, Winooski, VT, USA). Vc (40 μg/mL) was used as the positive control.

### 3.5. Hoechst 33258 Staining of B16 Cells

The B16 cells were treated with different concentrations of rFIP-glu (0, 6, 12, 24, 48 μg/mL) for 24 h, and washed with PBS and then stained with Hoechst 33258 for 30 min. After that, the cells were observed under a fluorescence microscope (Olympus, Tokyo, Japan), with an emission wavelength of 460 nm and an excitation wavelength of 350 nm.

### 3.6. Flow Cytometry Analysis

The B16 cells (4 × 10^4^/mL) were cultured for 24 h and treated with different concentrations of rFIP-glu (0, 6, 12, 24, 48 μg/mL) for 24 h, and then washed with PBS. The cells were adjusted to 1 × 10^6^ cells/mL with 1 × binding buffer, and 5 μL Annexin-V- Alexa Fluor 647 and 10 μL propidium iodide (PI) were added, prior to leaving for 15 min in the dark, according to the manufacturer’s instructions. After adding a 400 μL 1 × binding buffer, the cells were analyzed by a CytoFLEXS (Bechman coulter, Indianapolis, IN, USA) [34].

### 3.7. Determination of DPPH Free Radical Scavenging Activity

The detection was conducted according to the previous method. rFIP-glu were prepared with deionized water at 0, 0.5, 1, 2, 4 and 6 mg/mL, and 300 μL was added into each well of 96-well microtiter plates. After incubation in the dark for 10 min, the absorbance was measured at 517 nm with a microplate reader (BioTek, Winooski, VT, USA). Vc (0.5 mg/mL) was used as the positive control. The DPPH free radical scavenging rate was calculated as the following equation:

$$\mathrm{DPPH}\;\mathrm{free}\;\mathrm{radical}\;\mathrm{scavenging}\;\mathrm{rate}\;\left(\%\right)=\left(\mathrm{absorbance}\;\mathrm{of}\;\mathrm{control}-\mathrm{absorbance}\;\mathrm{of}\;\mathrm{sample}\right)/\mathrm{absorbance}\;\mathrm{of}\;\mathrm{control}\times 100$$

### 3.8. Determination of Melanin Production

A NaOH lysis assay was employed to determine the level of melanin synthesis of B16 cells [35,36]. B16 cells were seeded in a 6-well plate with a density of 4 × 10^4^ per well and incubated for 24 h. The culture was then replaced with different concentrations of rFIP-glu (0, 100, 500, 1000 μg/mL), and incubated for 72 h. A total of 200 μL NaOH (1M) solution containing 10% DMSO was added to each well, and the plate was maintained at 80 °C for 2 h. The absorbance was measured at 492 nm by a microplate reader (BioTek, Winooski, VT, USA). Arbutin (1000 μg/mL) was used as the positive control.

### 3.9. Determination of Tyrosinase Activity

B16 cells (4 × 10^4^/mL) were cultured for 24 h in a 12-well plate and then treated with different concentrations of rFIP-glu (0, 6, 12, 24, 48 μg/mL) for 48 h. An amount of 100 μL 1% Triton X-100 was added into each well and immediately frozen in −80 °C for 30 min. After melting at room temperature, samples were centrifuged at 12,000× *g* for 30 min at 4 °C, and the supernatant was collected. A total of 40 μL supernatant and 160 μL L-Dopa solution (2.5 mM) was mixed into a new 96-well plate and cultured at 37 °C for 1 h. The absorbance at 492 nm was measured by a microplate reader (BioTek, Winooski, VT, USA).

### 3.10. RT-qPCR Analysis

Total RNA was extracted from cells with the RNeasy Mini Kit. According to the manufacturer’s instructions, 1 μg RNA was synthesized into cDNA with PrimeScript ^TM^ RT Master Mix. After the cDNA was diluted 50 times, RT-qPCR was performed as the following program: 35 cycles of denaturing at 94 °C for 45 s, annealing at 62 °C for 45 s, extension at 72 °C for 1 min, and a final extension at 72 °C for 5 min. The primers used in the study are listed in Table 1. All reactions were performed in triplicate.

### 3.11. Statistical Analysis

Data were calculated as averages of experiments conducted in triplicate and expressed as means ± standard deviations (SD). A statistical analysis was performed by one-way analysis of variance (ANOVA) and a Student’s *t*-test (GraphPad Prism 8.0). Significance was indicated as NS, *p* > 0.05; *, *p* ≤ 0.05; **, *p* ≤ 0.01; ***, *p* ≤ 0.001; and ****, *p* ≤ 0.0001 versus the control group.

## 4. Conclusions

*P. pastoris* GS115 was employed for the production of rFIP-glu, but further experiments are still needed prior to large-scale production. rFIP-glu promoted the growth of HaCaT cells, while they induced the apoptosis of B16 cells. Meanwhile, rFIP-glu (6 mg/mL) had a similar DPPH free radical scavenging activity as vitamin C (0.5 mg/mL), and rFIP-glu (500 μg/mL) had a similar melanin synthesis inhibitory effect as arbutin (100 μg/mL), which is the active ingredient used in commercial whitening cosmetics. These data imply that rFIP-glu is a potential antioxidant and whitening agent. Furthermore, rFIP-glu functioned by initially stimulating levels of MITF and inhibiting the TYRP-1/TYR pathway, which thereby inhibited tyrosinase activity and melanogenesis. Therefore, this protein should be a strong candidate for treating melanin deposition in cosmeceutical applications. This study provided valuable insight into its suitability as a bioactive ingredient in products that repair skin damage and promote skin whitening.

## Figures and Tables

**Table 1:** Primers for RT-qPCR.

Genes	Direction	Sequence (5′ → 3′)
TYR	Forward	CACCATGCTTTTGTGGACAG
TYR	Reverse	GGCTTCTGGGTAAACTTCCAA
TRYP-1	Forward	CTGTGGATTATTGGGATGA
TRYP-1	Reverse	GTGAGCCACCACTTTGAG
TRYP-2	Forward	GCTGATTAGTCGGAACTCGA
TRYP-2	Reverse	GCTGATTAGTCGGAACTCGA
MITF	Forward	CAAATGGCAAATACGTTACCCG
MITF	Reverse	CAAATGGCAAATACGTTACCCG

## Data Availability

The data presented in this study are available on request from the corresponding author.

## Sample Availability

Samples of the compounds, including pPIC9K::FIP-glu-His and rFIP-glu, are available from the corresponding author.

## References

[1] Zhou X.-W., Su K.-Q., Zhang Y.-M. (2012). Applied modern biotechnology for cultivation of Ganoderma and development of their products. Appl. Microbiol. Biotechnol..

[2] Kino K., Yamashita A., Yamaoka K., Watanabe J., Tanaka S., Ko K., Shimizu K., Tsunoo H. (1989). Isolation and characterization of a new immunomodulatory protein, ling zhi-8 (LZ-8), from *Ganoderma lucidium*. J. Biol. Chem..

[3] Li Q.Z., Chang Y.Z., He Z.M., Chen L., Zhou X.W. (2019). Immunomodulatory activity of *Ganoderma lucidum* immunomodulatory protein via PI3K/Akt and MAPK signaling pathways in RAW264. 7 cells. J. Cell. Physiol..

[4] Li L.-D., Mao P.-W., Shao K.-D., Bai X.-H., Zhou X.-W. (2019). Ganoderma proteins and their potential applications in cosmetics. Appl. Microbiol. Biotechnol..

[5] Arung E.T., Furuta S., Ishikawa H., Tanaka H., Shimizu K. (2011). Melanin biosynthesis inhibitory and antioxidant activities of quercetin-3’-O-β-D-glucoside isolated from *Allium cepa*. Z. Für Nat. C.

[6] Arung E.T., Syafrizal Pasedan W.F., Tandirogang N., Sukemi Allam A.E., Amen Y., Shimizu K., Ishikawa H. (2020). Prenylated flavonoids as antioxidant and melanin inhibitors from stingless bee (*Wallacetrigona incisa*) propolis. Nat. Prod. Commun..

[7] Baswan S.M., Leverett J., Pawelek J. (2019). Clinical evaluation of the lightening effect of cytidine on hyperpigmented skin. J. Cosmet. Dermatol..

[8] Pakdel E., Xie W., Wang J., Kashi S., Sharp J., Zhang Q., Varley R.J., Sun L., Wang X. (2022). Superhydrophobic natural melanin-coated cotton with excellent UV protection and personal thermal management functionality. Chem. Eng. J..

[9] Liu J.-K. (2022). Natural products in cosmetics. Nat. Prod. Bioprospecting.

[10] Juhasz M.L., Levin M.K. (2018). The role of systemic treatments for skin lightening. J. Cosmet. Dermatol..

[11] Li Q., Wang X., Chen Y., Lin J., Zhou X. (2010). Cytokines expression induced by Ganoderma sinensis fungal immunomodulatory proteins (FIP-gsi) in mouse spleen cells. Appl. Biochem. Biotechnol..

[12] Mao P.-W., Li L.-D., Wang Y.-L., Bai X.-H., Zhou X.-W. (2020). Optimization of the fermentation parameters for the production of Ganoderma lucidum immunomodulatory protein by Pichia pastoris. Prep. Biochem. Biotechnol..

[13] Stoyneva-Gärtner M., Uzunov B., Gärtner G. (2020). Enigmatic microalgae from aeroterrestrial and extreme habitats in cosmetics: The potential of the untapped natural sources. Cosmetics.

[14] Levin J., Momin S.B. (2010). How much do we really know about our favorite cosmeceutical ingredients?. J. Clin. Aesthetic Dermatol..

[15] Thevanayagam H., Mohamed S.M., Chu W.-L. (2014). Assessment of UVB-photoprotective and antioxidative activities of carrageenan in keratinocytes. J. Appl. Phycol..

[16] Taofiq O., Rodrigues F., Barros L., Barreiro M.F., Ferreira I.C., Oliveira M.B.P. (2019). Mushroom ethanolic extracts as cosmeceuticals ingredients: Safety and ex vivo skin permeation studies. Food Chem. Toxicol..

[17] Cichorek M., Wachulska M., Stasiewicz A., Tymińska A. (2013). Skin melanocytes: Biology and development. Adv. Dermatol. Allergol..

[18] Osborne S.N., Schmidt M.A., Derrick K., Harper J.R. (2015). Epidermal micrografts produced via an automated and minimally invasive tool form at the dermal/epidermal junction and contain proliferative cells that secrete wound healing growth factors. Adv. Ski. Wound Care.

[19] Ali A., Gupta J. (2022). Applications of stem cell therapy and adipose-derived stem cells for skin repair. Curr. Dermatol. Rep..

[20] Merecz-Sadowska A., Sitarek P., Kowalczyk T., Zajdel K., Kucharska E., Zajdel R. (2022). The modulation of melanogenesis in B16 cells upon treatment with plant extracts and isolated plant compounds. Molecules.

[21] Jena K., Pandey J., Kumari R., Sinha A., Gupta V., Singh G. (2018). Free radical scavenging potential of sericin obtained from various ecoraces of tasar cocoons and its cosmeceuticals implication. Int. J. Biol. Macromol..

[22] Juang L.J., Gao X.Y., Mai S.T., Lee C.H., Lee M.C., Yao C.L. (2019). Safety assessment, biological effects, and mechanisms of *Myrica rubra* fruit extract for anti-melanogenesis, anti-oxidation, and free radical scavenging abilities on melanoma cells. J. Cosmet. Dermatol..

[23] Kim M., Shin S., Lee J.-A., Park D., Lee J., Jung E. (2015). Inhibition of melanogenesis by Gaillardia aristata flower extract. BMC Complement. Altern. Med..

[24] Watanabe F., Hashizume E., Chan G.P., Kamimura A. (2014). Skin-whitening and skin-condition-improving effects of topical oxidized glutathione: A double-blind and placebo-controlled clinical trial in healthy women. Clin. Cosmet. Investig. Dermatol..

[25] Kilala Tilaar M., Junardy F.D., Subroto E., Puspitosari D. (2018). Safety and efficacy evaluation on combination of *Lansium domesticum* fruit extract and *Hibiscus rosa-sinensis* flower extract as lightening agent for cosmetic. Int. J. Pharm. Med. Biol. Sci.

[26] Saeedi M., Khezri K., Seyed Zakaryaei A., Mohammadamini H. (2021). A comprehensive review of the therapeutic potential of α-arbutin. Phytother. Res..

[27] Wachamo S.A., Patel M.H., Varghese P.K., Dolinska M.B., Sergeev Y.V. (2021). Characterization of Temperature-Dependent Kinetics of Oculocutaneous Albinism-Causing Mutants of Tyrosinase. Int. J. Mol. Sci..

[28] Pillaiyar T., Manickam M., Namasivayam V. (2017). Skin whitening agents: Medicinal chemistry perspective of tyrosinase inhibitors. J. Enzym. Inhib. Med. Chem..

[29] Oh T.-I., Yun J.-M., Park E.-J., Kim Y.-S., Lee Y.-M., Lim J.-H. (2017). Plumbagin suppresses α-MSH-induced melanogenesis in B16F10 mouse melanoma cells by inhibiting tyrosinase activity. Int. J. Mol. Sci..

[30] Jeon G., Ro H.-S., Kim G.-R., Lee H.-Y. (2022). Enhancement of Melanogenic Inhibitory Effects of the Leaf Skin Extracts of Aloe barbadensis Miller by the Fermentation Process. Fermentation.

[31] Boo Y.C. (2021). Arbutin as a skin depigmenting agent with antimelanogenic and antioxidant properties. Antioxidants.

[32] Sun L., Guo Y., Zhang Y., Zhuang Y. (2017). Antioxidant and anti-tyrosinase activities of phenolic extracts from rape bee pollen and inhibitory melanogenesis by cAMP/MITF/TYR pathway in B16 mouse melanoma cells. Front. Pharmacol..

[33] Li Q.-Z., Chen X., Mao P.-W., Jin M.-Y., Wu Q., Zhou X.-W. (2021). N-Glycosylated *Ganoderma lucidum* immunomodulatory protein improved anti-inflammatory activity via inhibition of the p38 MAPK pathway. Food Funct..

[34] Liu Y., Li Q.-Z., Zhou X.-W. (2021). Immunostimulatory effects of the intracellular polysaccharides isolated from liquid culture of *Ophiocordyceps sinensis* (Ascomycetes) on RAW264. 7 cells via the MAPK and PI3K/Akt signaling pathways. J. Ethnopharmacol..

[35] Hong J.-H., Chen H.-J., Xiang S.-J., Cao S.-W., An B.-C., Ruan S.-F., Zhang B., Weng L.-D., Zhu H.-X., Liu Q. (2018). Capsaicin reverses the inhibitory effect of licochalcone A/β-Arbutin on tyrosinase expression in b16 mouse melanoma cells. Pharmacogn. Mag..

[36] Teng H., Fan X., Lv Q., Zhang Q., Xiao J., Qian Y., Zheng B., Gao H., Gao S., Chen L. (2020). *Folium nelumbinis* (Lotus leaf) volatile-rich fraction and its mechanisms of action against melanogenesis in B16 cells. Food Chem..

